# Ensemble Machine Learning- and Deep Learning-Driven Identification and Validation of Sennidin B as a Novel Dipeptidyl Peptidase-4 Inhibitor

**DOI:** 10.3390/ijms27125536

**Published:** 2026-06-18

**Authors:** Shahid Ali, Sibhghatulla Shaikh, Jeong Ho Lim, Eun Ju Lee, Inho Choi

**Affiliations:** 1 Department of Medical Biotechnology, Yeungnam University, Gyeongsan 38541, Republic of Korea; ali.ali.md111@gmail.com (S.A.); sibhghat.88@gmail.com (S.S.); lim2249@naver.com (J.H.L.); 2 Research Institute of Cell Culture, Yeungnam University, Gyeongsan 38541, Republic of Korea

**Keywords:** type 2 diabetes, dipeptidyl peptidase-4, artificial intelligence, deep learning, natural compounds, sennidin B

## Abstract

Dipeptidyl peptidase-4 (DPP-4) is a key therapeutic target for type 2 diabetes (T2D). Several synthetic anti-DPP-4 drugs are currently available for the treatment of T2D; however, the need for safe and effective therapies remains unmet due to the side effects associated with existing DPP-4 inhibitors. This study aimed to integrate structure-based and machine learning (ML)-based virtual high-throughput screening to identify natural DPP-4 inhibitors. Random forest, logistic regression, support vector machine (SVM), and multilayer perceptron (MLP) models were trained on DPP-4 IC_50_ datasets. Among these, the SVM and MLP models achieved high predictive performance, with areas under the curve of 0.928 and 0.923, respectively. Screening of a natural compound database identified 107 compounds for further analysis. Subsequent structure-based screening, using sitagliptin as a positive control, identified sennidin B and doxorubicin hydrochloride as promising candidates with strong binding affinity for DPP-4. Molecular dynamics simulations (200 ns) and MM-PBSA calculations confirmed stable interactions with DPP-4. Further, sennidin B and doxorubicin hydrochloride inhibited DPP-4 activity in a concentration-dependent manner, with estimated IC_50_ values of 39.39 and 19.78 μM, respectively. Sennidin B also reduced DPP-4 mRNA and protein expression levels in Caco-2 cells. Overall, sennidin B shows promise as a natural DPP-4 inhibitor and warrants further investigation as a potential antidiabetic agent.

## 1. Introduction

Diabetes mellitus is a metabolic disorder characterized by chronic hyperglycemia resulting from inadequate insulin secretion. Diabetes affects individuals of all ages and sexes and contributes substantially to global morbidity and mortality. Type 2 diabetes (T2D), which accounts for more than 90% of all cases, is characterized by hyperglycemia caused by dysfunction of pancreatic β-cells and insulin resistance, primarily in skeletal muscle and the liver [1].

Conventional diabetes treatments such as metformin, insulin, and sulfonylureas remain widely used for glycemic control [2]; however, these treatments are limited by numerous side effects, including hypoglycemia, organ toxicity, declining long-term efficacy due to resistance, and high costs, particularly in developing countries [3,4]. These limitations highlight the need for safer, more effective, and more affordable alternatives, including those derived from natural compounds.

Glucagon-like peptide-1 (GLP-1) and glucose-dependent insulinotropic polypeptide (GIP) are gut-derived metabolic hormones that lower blood glucose levels and help maintain glucose homeostasis. Deficiency or resistance to these hormones plays an important role in the development of T2D. GLP-1 and GIP are released in response to food intake, although the short half-lives of these hormones lead to rapid degradation by dipeptidyl peptidase-4 (DPP-4) [5]. DPP-4 inhibitors increase GLP-1 and GIP levels, thereby enhancing pancreatic β-cell insulin production and reducing postprandial and fasting hyperglycemia [6,7]. Although many DPP-4 inhibitors, such as gliptins, are available, the use of these inhibitors has been associated with adverse effects [8,9,10], underscoring the need to explore safer natural alternatives.

The drug discovery process is complex and time-consuming, involving several sequential stages. Drug candidates are initially evaluated for specificity and activity, followed by comprehensive pharmacokinetic and toxicological profiling [11]. Despite these assessments, many drugs fail later in development due to safety and efficacy concerns [12]. The advances in computational science, such as artificial intelligence (AI), provide alternative approaches for finding bioactive agents that target specific disorders. These techniques have the potential to accelerate drug development and lower failure rates [13,14]. Moreover, the use of AI has increased significantly, particularly in the pharmaceutical industry, where this technology facilitates the analysis of complex, multidimensional data. Notably, recent advances in AI have transformed applications in drug discovery and development [15].

**Figure 1:**
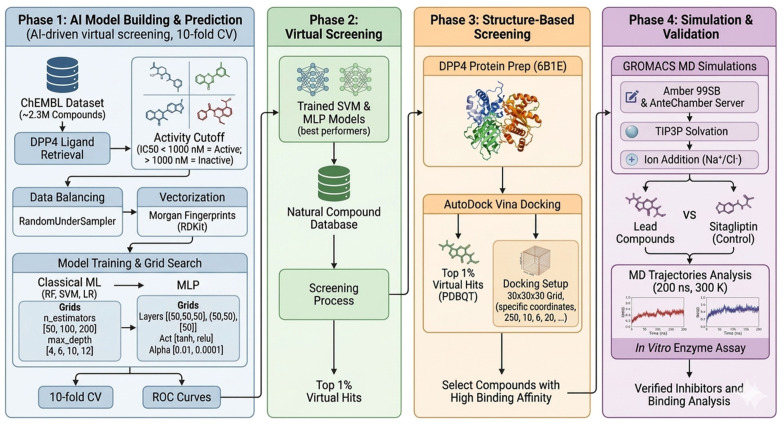
Study workflow. Experimental assays were performed to validate the results of AI-based and structure-based virtual screening and confirm the top hits.

Natural products are important sources of bioactive molecules for drug discovery, and several approved medications have been derived from these products [16]. Plant-derived compounds, in particular, have shown high therapeutic potential for metabolic disorders, including T2D [17,18,19,20]. Furthermore, we previously demonstrated that licochalcone A, licochalcone B, and isobavachalcone, identified through in silico screening, bind to key catalytic residues of DPP-4 and effectively inhibit enzymatic activity in vitro. Among these compounds, isobavachalcone showed significant in vivo efficacy by reducing high-fat diet-induced hyperglycemia while simultaneously enhancing circulating GLP-1 and insulin levels [21,22]. Therefore, this study aimed to find novel natural DPP-4 inhibitors for the treatment of T2D by integrating machine learning (ML) and deep learning approaches with in vitro validation (Figure 1).

## 2. Results and Discussion

### 2.1. Datasets Acquisition, ML Model Building, and Screening

**Figure 2:**
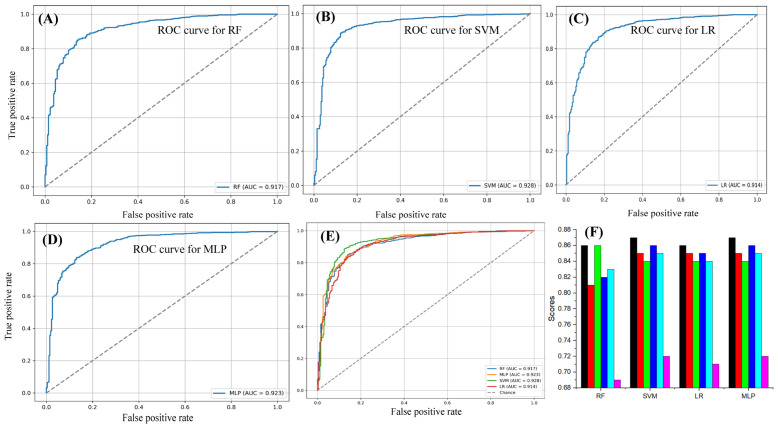
Evaluation of multiple models for screening natural compounds against DPP-4. (**A**) Random forest (RF), (**B**) support vector machine (SVM), (**C**) logistic regression (LR), (**D**) multilayer perceptron (MLP), (**E**) combined area under the curve (AUC) performance plot, and (**F**) data balanced matrix (bar color depicted black–precision, red–recall, green–specificity, blue–f1-score, cyan–geometric mean, and pink–index of balanced accuracy).

In this study, ML techniques were used as an initial screening strategy to assess natural compounds from the Biopurify and TargetMol databases. Bioactivity data for compounds targeting DPP-4 were obtained from the ChEMBL database. To improve data quality, redundant entries and duplicate compounds were identified and removed. The dataset included compounds with inhibitory activities at concentrations ranging from 0.05 nM to 9.1 × 10^6^ nM. Compounds with IC_50_ values less than 1000 nM were classified as active, while those with IC_50_ values exceeding 1000 nM were classified as inactive. Based on these criteria, the final dataset included 3344 active and 1438 inactive compounds. Molecular representations were generated through a vectorization process using Morgan fingerprints (1024-bit). Following vectorization, classical ML models, including random forest (RF), logistic regression (LR), support vector machine (SVM), and a multilayer perceptron (MLP) deep-learning classifier, were trained to discriminate between active and inactive compounds. Model performance and generalizability were assessed using 10-fold cross-validation, and receiver operating characteristic (ROC) curves were employed to visualize classification effectiveness. The mean area under the curve (AUC) values for the RF, LR, SVM, and MLP models were estimated at 0.91, 0.91, 0.92, and 0.92, respectively (Figure 2A–E). The trained model for the DPP-4 enzyme showed approximately 90% accuracy (Figure 2F). The natural compound database, which contains approximately 8796 compounds, was then used to identify potential DPP-4 hit compounds. Initially, fingerprints for all compounds were calculated using the RDKit tool (version 2025.9.2) and saved to a CSV file. Thereafter, screening was performed using the trained SVM and MLP models, with each model generating individual scores. After screening the compound database, all compounds were ranked based on the associated predicted scores from both trained models. To improve screening, the rankings of the SVM and MLP models were ensembled. Finally, the top 1% of hits from the screened database were selected based on the ensemble ranking. We also included the control inhibitor in the database to validate the accuracy of the top hits.

### 2.2. Structure-Based Screening of Top Hit Compounds

The ML models demonstrated strong performance in prioritizing natural compounds with potential inhibitory activity against DPP-4. Based on the prediction results, the top 1% of candidates (*n* = 107) were selected for subsequent structure-based virtual screening (VS). Three-dimensional molecular conformations were generated from their SMILES representations using RDKit and Open Babel, and the resulting structures were converted into PDBQT format to facilitate docking studies. Then, VS of the 107 compounds was conducted to find top hits with DPP-4 binding affinity scores greater than −8.0 kcal/mol. In molecular docking analysis, a more negative binding affinity value indicates a stronger interaction between the ligand and the associated target protein [23,24]. Finally, the two top compounds, sennidin B and doxorubicin hydrochloride (doxorubicin HCl), were selected, with binding affinities of −9.8 and −8.7 kcal/mol, respectively (Table 1). These compounds exhibited greater binding affinity than sitagliptin. Sennidin B and doxorubicin HCl were evaluated for the associated drug-likeness properties. The compounds violated the Lipinski rule of five, which is acceptable for natural compounds [25,26], and showed no mutagenic or carcinogenic properties (Table S1).

**Figure 3:**
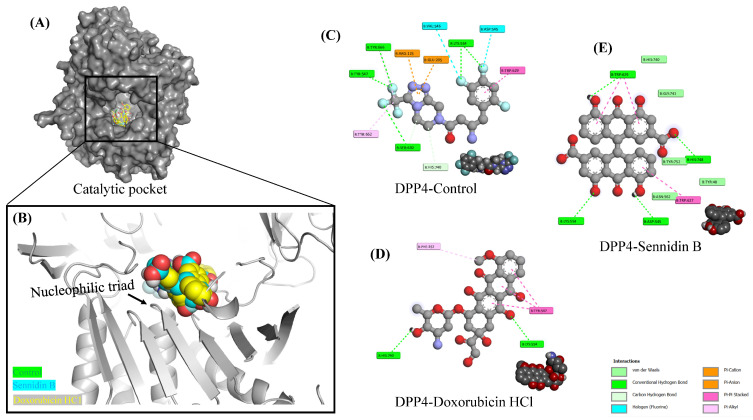
Residue-interaction analysis of DPP-4 with compounds. (**A**,**B**) Structural alignment of sennidin B and doxorubicin HCl, along with the control (sitagliptin), in the catalytic pocket of DPP-4. (**C**–**E**) Interacting residues of DPP-4 with sennidin B, doxorubicin HCl, and sitagliptin.

Moreover, an interaction analysis of sennidin B and doxorubicin HCl with the DPP-4 binding pocket was conducted and compared with that of the positive control, sitagliptin. The structural alignments of both hits (sennidin B and doxorubicin HCl) and the control (sitagliptin) overlapped and shared similar structural orientations in the catalytic pocket of DPP-4 (Figure 3A,B). Sitagliptin demonstrated four H-bond interactions with the DPP-4 residues SER-630, TYR-666, TYR-547, and LYS-554, as well as halogen interactions with VAL-546 and ASP-545 (Figure 3C). Sennidin B formed four hydrogen bond interactions with the ASP-545, LYS-554, TRP-629, and HIS-748 residues of DPP-4 (Figure 3D). Doxorubicin HCl showed two hydrogen bond interactions with the LYS-554 and HIS-740 residues of DPP-4 (Figure 3E). Interaction analysis showed that the DPP-4–sennidin B complex exhibited stronger interactions within the DPP-4 binding pocket and shared several interacting residues with sitagliptin. The active-site residues involved in DPP-4 inhibition are TYR547, SER630, TYR631, VAL656, TRP659, TYR662, TYR666, ASN710, VAL711, HIS740, GLU205, GLU206, TYR662, SER209, ARG358, and PHE357 [27]. Notably, sennidin B interacted with most of the key active-site residues of DPP-4.

### 2.3. Dynamic Stability Studies in the Catalytic Pocket of DPP-4

**Figure 4:**
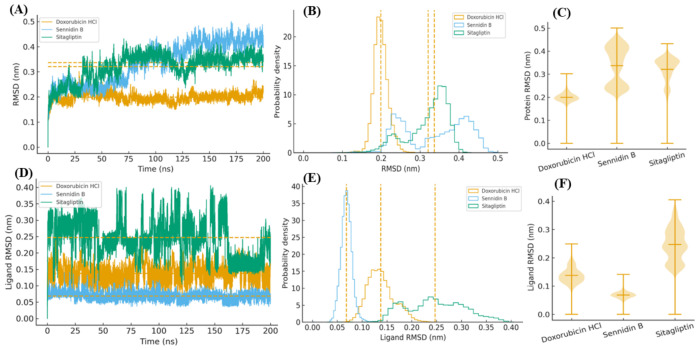
Dynamic conformational stability analysis of the hits. (**A**,**D**) Root-mean-square deviation (RMSD) of the DPP-4 backbone, (**B**,**E**) probability density of conformations, and (**C**,**F**) violin plots.

**Figure 5:**
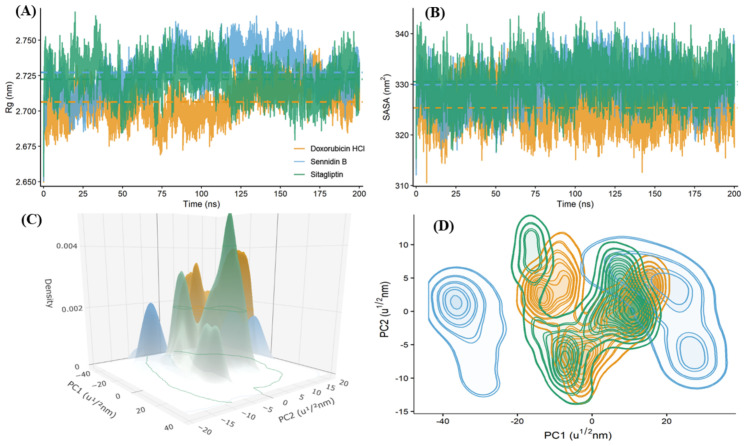
Conformational clustering of hits. (**A**) Radius of gyration of the DPP-4 backbone, (**B**) SASA of DPP-4, and (**C**,**D**) PCA analysis of complexes.

A 200 ns MD simulation was used to assess the stability of these compounds in complex with DPP-4, enabling a detailed evaluation of the associated thermodynamic stability and binding interactions. The DPP-4–doxorubicin HCl complex was noted for maintaining the highest level of protein structural integrity; the narrow probability density peak of the complex at 0.2 nm and minimal deviation in the violin plots suggest that the protein remained in a highly stable, near-native state throughout the simulation (Figure 4A–C). In contrast, the DPP-4–sitagliptin and DPP-4–sennidin B complexes induced larger conformational shifts in the protein, which could imply a significant induced-fit mechanism or less optimal stabilization of the secondary and tertiary structures. Regarding the behavior of the ligands within the binding pocket (Figure 4D–F), DPP-4–sennidin B demonstrated exceptional “locking” capability. The ligand root-mean-square deviation (RMSD) for this complex remained remarkably low and flat (<0.1 nm), indicating that the complex formed a highly rigid network of intermolecular interactions that prevented the complex from rotating or shifting within the active site. Sitagliptin showed the highest degree of flexibility, with the associated multimodal distribution (Figure 4E), suggesting that this compound explored several distinct sub-orientations during the 200 ns simulation. Doxorubicin HCl was slightly more mobile than sennidin B, but the associated ligand RMSD was more consistent than that of sitagliptin, indicating greater stability within the pocket. Throughout the simulation, all complexes maintained constant Rg values (~2.65–2.75 nm), suggesting the protein’s structural compactness, and it showed that there were no major conformational changes upon ligand binding. In addition, SASA measures the degree of protein surface exposure to solvent; steady SASA profiles over the whole simulation demonstrate that none of the ligands showed significant protein unfolding or exposure of hydrophobic core regions (Figure 5A,B). DPP-4–Doxorubicin HCl and DPP-4–sitagliptin occupied well-defined energy minima with compact conformational clusters, indicating stable binding, whereas DPP-4–Sennidin B exhibited broader density distributions and multiple spatially separated clusters, implying greater conformational flexibility and diverse DPP-4 binding modes (Figure 5C,D). Further, MM-PBSA binding free energy calculations of DPP-4–sennidin B, DPP-4–Doxorubicin HCl, and DPP-4–sitagliptin complexes were −35.89, −22.85, and −9.25 kcal/mol, respectively. It is also shown that screened leads exhibited better stability with DPP-4 (Table S2). Overall, both test compounds demonstrated superior stability compared with the control. Notably, sennidin B exhibited the greatest individual ligand stability, whereas doxorubicin HCl produced the most stable overall protein–ligand complex.

### 2.4. In Vitro DPP-4 Enzyme Assay

**Figure 6:**
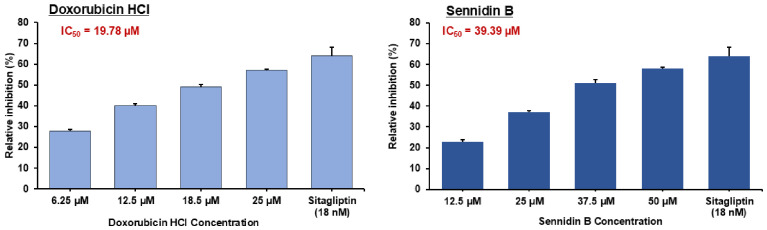
Percentage inhibition of DPP-4 by sennidin B and doxorubicin HCl and the corresponding IC_50_ values (*n* = 3).

The ability of sennidin B and doxorubicin HCl to inhibit DPP-4 activity was assessed at different inhibitor concentrations. Sennidin B and doxorubicin hydrochloride inhibited DPP-4 activity in a concentration-dependent manner, with estimated IC_50_ values of 39.39 and 19.78 μM, respectively (Figure 6, Figure S1). Doxorubicin HCl inhibited DPP-4 activity with an IC_50_ lower than that of sennidin B; however, the clinical use of doxorubicin HCl is limited due to the established toxicity profile of the drug. Indeed, doxorubicin, an anthracycline chemotherapeutic commonly used to treat various cancers, is associated with a high risk of cardiotoxicity, which can lead to heart failure and death [28,29,30,31]. Therefore, despite the higher in vitro DPP-4 inhibitory potency, doxorubicin HCl was excluded from further in vitro investigations in the present study, and sennidin B was selected for subsequent in vitro evaluation.

### 2.5. Binding of Sennidin B to DPP-4 and Its Effect on DPP-4 Expression in Sennidin B-Treated Caco-2 Cells

**Figure 7:**
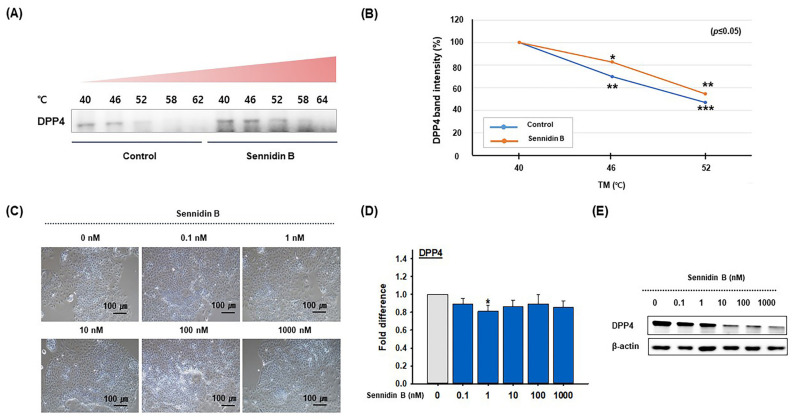
Binding of sennidin B to DPP-4 and DPP-4 expression after sennidin B treatment. DPP-4 protein levels were measured in Caco-2 cells treated with dimethyl sulfoxide (DMSO; control) or 1000 nM sennidin B and heated at 40, 46, 52, 58, and 64 °C. Protein levels were assessed by Western blot; temperature-dependent changes in DPP-4 intensity were noted between control and sennidin B-treated cells. (**A**) DPP-4 protein intensity by Western blot. (**B**) DPP-4 protein intensity quantified using ImageJ (version 1.54). Sennidin B (0, 0.1, 1, 10, 100, and 1000 nM) was administered to Caco-2 cells for 4 days. (**C**) Cell morphology (**D**) DPP-4 mRNA expression by real-time RT-PCR. (**E**) DPP-4 protein expression by Western blot. Untreated cells were used as the control (mean ± standard deviation (SD), *n* ≥ 3; * *p* ≤ 0.05, ** *p* ≤ 0.001, *** *p* ≤ 0.0001).

The therapeutic potential of a compound is closely associated with its ability to engage its molecular target. Cellular thermal shift assay (CETSA) provides a reliable validation of the binding of the compound to the target protein [22,32], demonstrating the overall suitability of the compound for confirming ligand–target interactions. In this study, CETSA was employed to validate the interaction between sennidin B and DPP-4. Caco-2 cells were treated with 1000 nM sennidin B and subjected to thermal challenge. Compared with untreated controls, cells treated with sennidin B exhibited significantly higher DPP-4 protein intensity across the thermal challenge (Figure 7A,B), indicating that sennidin B binds to DPP-4 and enhances its thermal stability.

Caco-2 cells are a well-known intestinal epithelial model because of their ability to mimic key structural and functional characteristics of small intestinal enterocytes [33]. This cell line is especially useful for evaluating compounds that modulate DPP-4 expression because it has high levels of *DPP-4* mRNA expression and apically localized active DPP-4 proteins [34,35]. Caco-2 cells were treated with sennidin B at concentrations of 0, 0.1, 1, 10, 100, and 1000 nM for 4 days. DPP-4 mRNA expression was significantly decreased only at 1 nM compared with the control group, whereas no consistent dose-dependent decrease was observed at higher concentrations (Figure 7C,D). In contrast, DPP-4 protein expression showed an overall decreasing trend with increasing concentrations of sennidin B (Figure 7E). Collectively, these results indicate that sennidin B suppresses both DPP-4 mRNA and protein expression.

Sennidin B was identified through an integrated AI and in silico screening approach, highlighting the growing significance of computational approaches in natural product-based drug discovery. Natural products are a rich source of structurally diverse and biologically active compounds [16]. However, compared to clinically approved synthetic DPP-4 inhibitors, many naturally occurring compounds have weaker inhibitory activity, often in the micromolar concentration range. Previous studies demonstrate that natural products such as flavonoids and phenolic derivatives have significantly weaker DPP-4 inhibitory activity than synthetic gliptins [36,37]. For instance, citrus bioflavonoids demonstrated IC_50_ values ranging from 485 μM to 5700 μM against DPP-4, which are several orders of magnitude less potent than sitagliptin (IC_50_ = 0.684 μM) [36]. Although sennidin B exhibited weaker inhibitory potency than the approved DPP-4 inhibitor sitagliptin, its favorable target-binding characteristics and natural-product scaffold provide a promising starting point for hit-to-lead optimization. Previously, we developed AI-assisted in silico platforms to identify DPP-4 inhibitors and other bioactive compounds targeting metabolic pathways, demonstrating that combining ML and molecular docking enables the efficient detection of high-affinity DPP-4 inhibitors [38]. Meanwhile, this approach was extended in the present study and identified sennidin B as a high-confidence candidate that inhibited DPP-4 enzyme activity in vitro and suppressed both DPP-4 mRNA and protein expression at the cellular level. These findings support the use of AI-assisted natural product screening to bridge the gap between chemical diversity and biological activity [39].

## 3. Materials and Methods

### 3.1. AI Model Building and Prediction

A curated dataset of DPP-4 ligands was compiled from publicly available chemical databases, primarily ChEMBL, to identify compounds with experimentally determined activity data. The activity classification was based on IC_50_ values, with a threshold of 1000 nM used to distinguish active inhibitors from inactive compounds. Molecular vectorization was then used to encode chemical structures in machine-readable formats. RDKit was used to create molecular fingerprints that served as structural descriptors for training predictive models, evaluating their performance, and analyzing the chemical properties of the compounds. All circular fragments were counted from each selected heavy-atom center up to a specified radius of two atoms, using 1024-bit Morgan FPs [40]. Get_morganfp was used to perform the FP computations. The ML models were developed using standard Python (3.9) packages. Four approaches were selected to represent ML algorithms: RF, LR, SVM, and MLP, a type of neural network. The scikit-learn [41] package was used to implement the models. The RandomUnderSampler function from the imblearn.under_sampling function was used to address the class imbalance between active and inactive compounds. GridSearchCV [41] was used to identify the optimal hyperparameter settings for the trained models. The classical ML (RF, LR, and SVM) hyperparameter grid included n_estimators (50, 100, and 200) and max_depth (4, 6, 10, and 12). The MLP hyperparameter grid included hidden layer sizes [(50, 50, 50), (50, 50), and (50)], activation functions (tanh and relu), and alpha values (0.01 and 0.0001). The trained models were evaluated using ten-fold cross-validation, and ROC curves were plotted for each model. Subsequently, the ensemble strategy was employed based on rank averaging. Specifically, after independent VS using SVM and MLP models, each compound was assigned a rank based on its predicted probability score in descending order. The ensemble rank for each compound was then calculated as the arithmetic mean of its SVM rank and MLP rank:

Ensemble Rank = (SVM_rank + MLP_rank)/2

Finally, the top 1% of compounds identified through virtual screening of the natural compound library were selected for further analysis.

### 3.2. DPP-4 Protein Preparation and Structure-Based Screening

The three-dimensional (3D) structure of DPP-4 (PDB ID: 6B1E) was retrieved from the Protein Data Bank. The DPP-4 protein structure was created by removing heteroatoms and water molecules, and the final structure was saved as a pdb file. AutoDock Vina (version 1.2.7) was used to perform the molecular docking experiment, and PDBQT files were generated from the PDB structures of proteins and ligands for docking analysis. The catalytic pocket was defined based on the key nucleophilic residues of DPP-4 (Ser-630, Asp-708, and His-740), with the grid box centered at X = −0.022, Y = 60.774, and Z = 37.925. The docking results were calculated and ranked using negative values, with more negative values indicating higher binding affinities. Compounds with binding affinities higher than that of sitagliptin were selected for further study.

### 3.3. Molecular Dynamics Simulation and MM-PBSA Binding Free Energy Calculations

GROMACS 2022 was used to perform MD simulations of the hit compounds and sitagliptin in complex with DPP-4 at 300 K [42]. The AMBER99SB force field was used to determine the topology of the DPP-4 enzyme. The AnteChamber (version 1.27) server was used to generate the topologies and force-field parameters of the hit compounds [43]. The TIP3P water model was used to solvate the hit–DPP-4 complex in a cubic box [44]. To restore charges on the hit–DPP-4 complexes, Na^+^ and Cl^−^ ions were added using the gmx genion module at a physiological concentration of 0.15 M. Each system was energy-minimized using 1500 steps of the steepest descent approach. MD simulations were run for 200 ns per hit–DPP-4 complex, and the resulting trajectories were used for further investigation. The 3D models were graphically represented using VMD (version 1.9.3) and PyMOL (version 3.1.8). Binding free energies were calculated using the Molecular Mechanics Poisson–Boltzmann Surface Area (MM-PBSA) method, which was implemented in gmx_MMPBSA v1.6.3 [45], which is based on MMPBSA.py v16.0 [46]. The estimates were based on 500 frames collected at consistent intervals from the production trajectory. The binding free energy was calculated based on:

ΔG_bind = ΔG_complex − ΔG_receptor − ΔG_ligand

The term ΔG_complex refers to the complex’s absolute free energy, whilst ΔG_receptor and ΔG_ligand signify the absolute free energy measurements for the DPP-4 enzyme and ligands (control, sennidin B, and doxorubicin HCl), respectively.

### 3.4. DPP-4 Enzyme Assay

A DPP-4 Inhibitor Screening kit (Sigma-Aldrich, St. Louis, MO, USA) was used to validate the inhibitory activity of selected compounds following the manufacturer’s protocols. Relative percentage of DPP-4 inhibition was performed as described previously [21].

### 3.5. Caco-2 Cell Culture

Caco-2 cells obtained from ATCC (Manassas, VA, USA) were cultured in MEM (Cytiva, Marlborough, MA, USA) supplemented with 10% FBS (Cytiva), 1% penicillin-streptomycin (Cytiva), 1% GlutaMAX (Thermo Fisher Scientific, Waltham, MA, USA), and 1% MEM non-essential amino acids (Thermo Fisher Scientific). Cell cultures were maintained at 37 °C in a humid incubator with 5% CO_2_.

### 3.6. Sennidin B Treatment

To evaluate the effects of sennidin B (Biopurify Phytochemicals Ltd., Chengdu, China; molecular formula C_30_H_18_O_10_; molecular weight 538.464 g/mol), Caco-2 cells were cultured in growth medium containing sennidin B at final concentrations of 0, 0.1, 1, 10, 100, or 1000 nM for 4 days.

### 3.7. Cellular Thermal Shift Assay

Caco-2 cells were treated with either DMSO (vehicle control) or 1000 nM sennidin B for 2 h at 37 °C in a 5% CO_2_ incubator. After treatment, cells were collected, washed three times with PBS, and resuspended in PBS containing protease inhibitor cocktail (Thermo Fisher Scientific). Samples were exposed to a temperature gradient ranging from 40 to 64 °C for 3 min using a thermal cycler to assess protein thermal stability. Cell lysis was achieved by repeated freeze–thaw cycles in liquid nitrogen, followed by centrifugation to isolate soluble proteins. The thermal stability of DPP-4 was subsequently evaluated by Western blotting with an anti-DPP-4 antibody (Cell Signaling Technology, Danbury, MA, USA).

### 3.8. RNA Isolation and Real-Time RT-PCR

Total RNA was extracted, and real-time RT-PCR was performed as previously described [47]. Primer sequences used for amplification are listed in Table S3.

### 3.9. Western Blot

Protein samples (100 µg for Caco-2 cells) were resolved by sodium dodecyl sulfate-polyacrylamide gel electrophoresis and subsequently transferred onto polyvinylidene difluoride membranes. Membranes were blocked with an appropriate blocking solution and incubated overnight at 4 °C with primary antibodies diluted in 1% bovine serum albumin in Tris-buffered saline. The primary antibodies used were anti-DPP-4 (1:1000) and anti-β-actin (1:1000; Santa Cruz Biotechnology, Dallas, TX, USA). Following washing, membranes were incubated with horseradish peroxidase-conjugated secondary antibodies (goat anti-rabbit or goat anti-mouse; GeneTex, Irvine, CA, USA) at room temperature for 1 h. Protein bands were detected using SuperSignal West Pico Chemiluminescent Substrate (Thermo Fisher Scientific).

### 3.10. Statistical Analysis

Normalized mean values were compared using Tukey’s post hoc test following one-way analysis of variance (ANOVA) performed in SAS software (version 9.0; SAS Institute, Cary, NC, USA). GAPDH was used as an internal control gene for normalization. Significance was assigned at two-tailed *p* values of <0.05, <0.01, or <0.0001.

## 4. Conclusions

This investigation employed a combined ML and structure-based strategy for the high-throughput VS of natural compounds against DPP-4. Sennidin B was identified as a potent DPP-4 inhibitor with favorable binding affinity and dynamic stability, interacting with the DPP4’s catalytic site and exhibiting interaction patterns similar to sitagliptin at several key residues. Further, in vitro investigations revealed that sennidin B inhibited DPP-4 activity in a concentration-dependent manner and reduced DPP-4 mRNA and protein expression in Caco-2 cells. These findings highlight the potential of sennidin B as a novel natural DPP-4 inhibitor for the management of T2D. However, additional in vivo investigations are required to establish its antidiabetic efficacy and effects on glycemic control.

## Figures and Tables

**Table 1:** Compounds screened using ML and structure-based virtual screening approaches.

Compound	MolWt (g/mol)	MolLogP	NumHAcceptors	NumHDonors	MLP Prediction Score	MLP Rank	SVM Prediction Score	SVM Rank	Ensemble Rank	Binding Affinity (Kcal/mol)
Sennidin B	538.464	3.9582	8	6	0.99	336	0.66	116	226	−9.8
Doxorubicin HCl	543.525	0.0013	12	6	0.99	283	0.65	253	268	−8.7
Sitagliptin	407.318	2.0165	5	1	0.99	237	0.95	1	119	−8.0

## Data Availability

The original contributions presented in the study are included in the article/Supplementary Material; further inquiries can be directed to the corresponding authors.

## Supplementary Materials

The following supporting information can be downloaded at: https://www.mdpi.com/article/10.3390/ijms27125536/s1.
