# Supplementary material for: Ensemble Machine Learning- and Deep Learning-Driven Identification and Validation of Sennidin B as a Novel Dipeptidyl Peptidase-4 Inhibitor
Source: Int J Mol Sci. 2026 Jun 18;27(12):5536. doi: 10.3390/ijms27125536 (PMC13300722; doi:10.3390/ijms27125536)
Supplement: Supplementary file 1 [file ijms-27-05536-s001.zip › ijms-4358388-supplementary.pdf]

Supplementary Table S1. Drug likeness properties of selected compounds.

Name	Formula	MW (g/mol)	TPSA	Ali Log S	GI absorption	BBB permeant	Pgp substrate	CYP1A2 inhibitor	CYP2C19 inhibitor	CYP2D6 inhibitor	CYP3A4 inhibitor	log Kp (cm/s)	Lipinski #violations	PAINS #alerts	Mutagenic
**Sennidin B**	$C_{30}H_{18}O_{10}$	538.464	189.66	-8.52	Low	No	No	No	No	No	No	-6.18	2	0	None
**Doxorubicin HCl**	$C_{27}H_{29}NO_{11}$	543.52	206.07	-5.2	Low	No	Yes	No	No	No	No	-8.71	3	1	None

Name	CNS permeability	Renal OCT2 substrate	hERG I inhibitor	hERG II inhibitor	Hepatotoxicity	Skin Sensitisation
**Sennidin B**	-3.661	No	No	No	No	No
**Doxorubicin HCl**	-4.317	No	No	No	No	No

Supplementary Table S2. Binding free energy calculation of the stable region of DPP-4 complexes.

Compound	ΔVDWaals	ΔEEL	ΔEPB	ΔENPOLAR	ΔGGAS	ΔGSOLV	ΔG_{bind} (kcal/mol)	Binding affinity (Kcal/mol)
**Sennidin B**	-40.87	-67.91	+76.80	-3.91	-108.78	+72.89	-35.89 ± 4.15	-9.8
**Doxorubicin HCl**	-41.65	-14.54	+37.00	-3.67	-56.19	+33.33	-22.86 ± 3.62	-8.7
**Sitagliptin**	-17.33	-8.44	+18.21	-2.19	-25.77	+16.03	-9.75 ± 3.94	-8.0

Supplementary Table S3. Primer information.

Primer	Size (bp)	Tm (°C)	Forward	Reverse
**GAPDH**	**184**	**59**	5'-gagtcaacggatttggtcgt-3'	5'-ttgattttggagggatctcg-3'
**DPP4**	**236**	**59**	5'-atgtccagatgccctccaaa-3'	5'-accacttcctctgccatcaa-3'

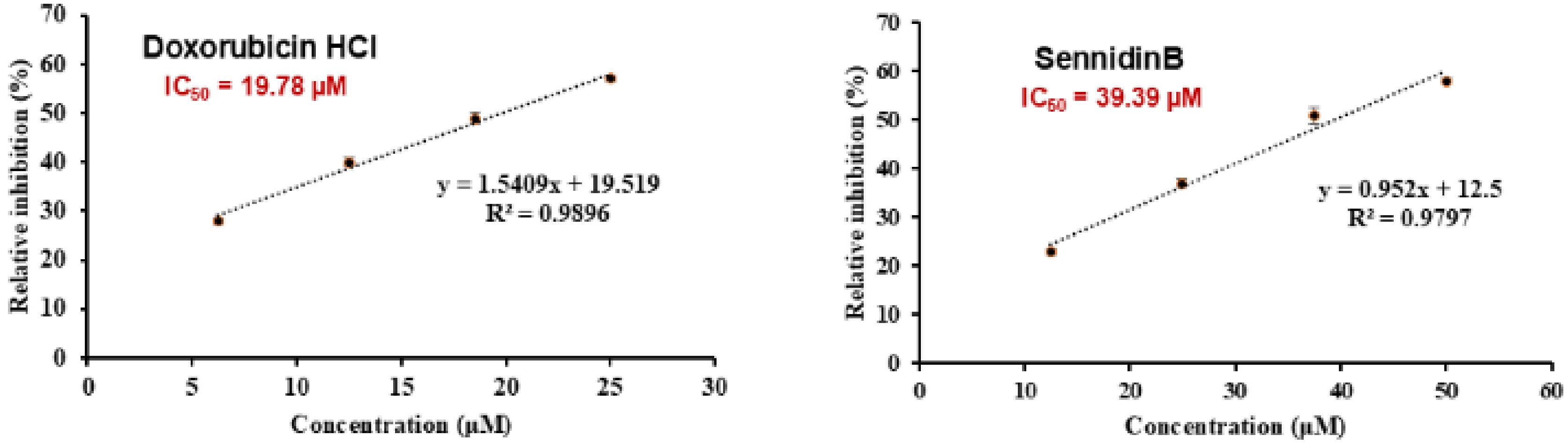

Supplementary Figure S1. IC_{50} values of doxorubicin HCl and sennidin B (n = 3).
